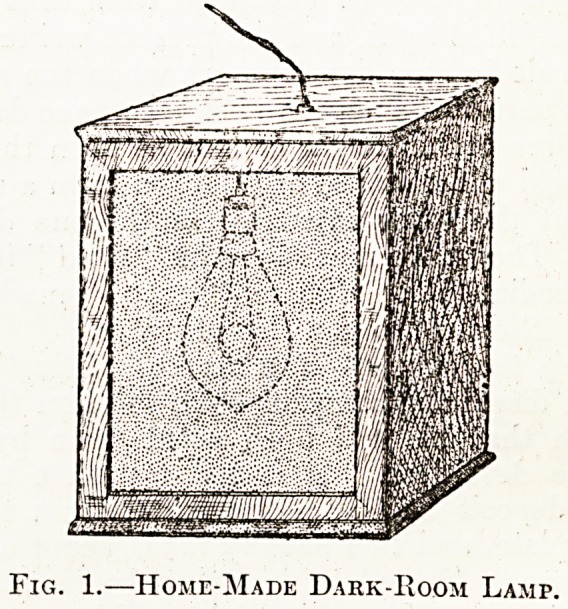# Radiography

**Published:** 1913-01-11

**Authors:** Alfred C. Norman

**Affiliations:** House Surgeon at Durham County Eye Infirmary.


### ELECTRICITY IN MODERN MEDICINE.*

### XXIV. Radiography (*Continued*).

By ALFRED C. NORMAN, M.D. Edin., House Surgeon at Durham County Eye Infirmary.

### Some Further Photographic Considerations.

*Plate Holders.*—Tylar's light-tight envelopes are generally used to protect the photographic plate from daylight while the radiographic exposure is being made. The plate is placed in a yellow envelope, with its film side opposite the smooth side of the envelope; the yellow envelope is then enclosed in a black one, so that the film side of the plate is opposite the smooth side of the black envelope. By this method we can always be sure of having the film side of the plate uppermost when radiographing a patient—and this is a matter of considerable importance when it is remembered that glass of the thickness used in these plates is very opaque to $x$-rays.

A light-proof cassette is sometimes used instead of envelopes for holding the photographic plate during the exposure. A radiographic cassette resembles an ordinary dark slide, the front being made of some thin material that will not obstruct the $x$-rays, but it has the disadvantage that it must be taken to the dark room every time an exposure is made, whereas when envelopes are used a dozen of them may be kept at hand ready filled with plates. For stereoscopic radiography, however, a special cassette is necessary in order that we may remove the plate and insert another without changing the patient's position in the slightest degree.

*Support for the Plate.*—The photographic plate, in its envelopes, should be supported on a flat board covered with sheet lead $\frac{1}{16}$ of an inch thick. This serves two purposes: it saves the plate from being cracked by the weight of the patient, as it would be if it were resting on an irregular surface; and it prevents the generation of secondary rays in the material behind the plate. If the reader doubt the possibility of this let him make an exposure of some object with half the plate resting on plain wood and the other half on sheet lead, and compare the two sides. After passing through the plate a certain number of rays traverse the wood and generate secondary rays in it, which tend to strike back and fog the former in a manner somewhat analogous to halation in ordinary photography, whereas the backing of sheet lead completely stops the rays and so prevents this.

*The Dark-Room Lamp.*—Red light exerts very little chemical action upon a photographic plate, consequently it is always used in a dark room where uncovered plates are manipulated, but it should be remembered that even red light affects the plate to a certain extent, hence they should be exposed to it as little as possible. A satisfactory red lamp can be made by any joiner for a few shillings. It consists of a wooden box, 9 by 7 by 7 inches, with slots in front to carry two pieces of dark ruby glass $8\frac{1}{2}$ by $6\frac{1}{2}$ inches square, and with a fitting inside for an ordinary 16 c.p. frosted carbon electric lamp; all joints being made perfectly light-tight. By removing the ruby glass and inserting a sheet of ground glass in the slots such a box may also be used as a viewing box for whole-plate negatives.

### Developing the Negative.

The film of a photographic plate consists of a gelatine emulsion containing bromide of silver. After an exposure has been made the film appears to the eye to be quite unchanged. But, as a matter of fact, an invisible chemical action exerted by the $x$-rays or by ordinary light in the case of photography) has rendered the silver

Fig. 1.—Home-Made Dark-Room Lamp.

bromide *capable of being reduced* to black metallic silver by certain chemical agents popularly termed *developers*. Development, then, consists in converting by chemical means a latent image of silver bromide into a visible image of black metallic silver. The amount of chemical action that can take place (and consequently the density of the image) depends upon the quantity of $x$-rays that have been allowed to reach the plate; hence it is that dense parts of the body (such as the bones, that prevent most of the rays from reaching the plate) are represented by almost transparent film, whereas the more transparent tissues are represented by blackened film—in other words, the image on the radiographic plate is a *negative* one, in so far as relative densities are concerned, though the relative positions of the parts are shown the right way round. It is a simple matter to make a *positive* print from the negative; and in this, of course, the dense parts of the body will appear dark and the transparent parts light, just as they do on the fluorescent screen, but a certain amount of detail will be lost in the printing pro-

* Previous articles appeared on Nov. 11, 25, Dec. 9, 30, 1911, Jan. 13, 27, Feb. 17, March 9, 30, April 20, May 4, 25, June 8, July 6, Aug. 3, 17, 31, Sept. 28, Oct. 12, 26, Nov. 9, 30, and Dec. 14, 1912.

cess, hence it is advisable to make the diagnosis from the negative whenever possible. A further disadvantage of the positive radiograph is that the position of the parts is reversed, so that the right side of the body appears to be the left and *vice versa*.*

### The Developer.

A metol-hydroquinone developer is best for *x*-ray work and the Ilford one-solution formula leaves nothing to be desired. The manipulations are as follows: The developing dish should be warmed, the photographic plate should be placed film upwards in the developer, and the dish should be rocked gently from side to side in order to ensure that no air bubbles or particles of sediment remain on the surface of the plate. After a few seconds the parts most affected by the rays will begin to blacken and gradually the whole picture will appear. It is impossible, however, to judge when development has gone far enough by merely looking at the *surface*, for the *x*-rays have passed right through the emulsion, and have affected its deeper parts as well. The negative must be examined from time to time by *transmitted* light, and the present writer finds it a safe rule to stop development when the most important part of the negative has become so dense that the outline of his finger cannot be seen through it when held at a distance of 6 inches from a red light of about the same intensity as the one described above. The image must then be " fixed " in a solution of sodium thio-sulphate (" hypo ") made by dissolving 12-oz. hypo in a pint of water.

*Fixing the Image.*—The process of fixation consists in dissolving away in hypo solution the silver bromide that has not been acted upon by *x*-rays and the developer. It is not complete until the milky deposit has all disappeared from the back of the plate, and this usually takes about a quarter of an hour. The plate may then be removed from the dark room (for white light will have no action upon the metallic silver image that remains), washed for an hour in running water, and carefully dried.

*The Importance of Warming the Developer.*—Warming the developer markedly increases its chemical activity, consequently the time of development may be reduced and the radiographic exposure considerably shortened by this means. The standard exposures given on page 246 were worked out with a developer warmed to 78° F.

Of course, the developer must not be too warm or it may blister the gelatine film or even dissolve it. Ilford plates will stand a temperature of nearly 80° F. without blistering, and if the porcelain developing dish be rinsed out with boiling water before the developer is poured in it will retain sufficient heat to raise the latter to the required temperature without further trouble.

*The Evils of Over-exposure.*—Under-exposure may to a small extent be compensated for by prolonging the time of development, but over-exposure must necessarily be fatal to the quality of the radiograph; and yet over-exposure is by far the most common fault in radiography. An over-exposed radiograph presents a flat, muddy appearance, with very little contrast between the faintly outlined image and the rest of the plate. This appearance to the beginner suggests under-exposure, and so he goes on increasing the length of his exposures, and probably the penetration of his tube as well, until his technique reaches a state of absolute chaos.

### The Use of Intensifying Screens.

An intensifying screen is a sort of fluorescent screen in which the grain has been reduced to a minimum. It consists of a thin sheet of cardboard coated with some chemical that becomes luminous under the influence of the *x*-rays. The coated side of the screen is placed in close contact with the film side of the photographic plate, and its degree of luminosity is in proportion to the quality of *x*-rays that pass through it. The result is that a photochemical effect is added to the radio-chemical effect taking place in the film of the plate, and consequently the exposure may be reduced to about one-fifth of the standard. Unfortunately, even the so-called grainless types of screen somewhat coarsen the texture of the radiograph, consequently their use has not become general. In radiographing the heart, or the stomach and intestines after a bismuth meal, a little coarsening is no great detriment compared with the enormous advantage obtained by reducing the exposures, and it is in these branches of radiography that the newer types of intensifying screen promise to be most valuable.

The writer feels that he cannot conclude this section without expressing his obligation to Mr. W. E. Schall, B.Sc. Mr. Schall has done a good deal of experimental work in order to confirm the constant value of the milliampere-second and other *x*-ray units, and he has always been most willing to place his results at the writer's disposal.

Space does not permit of my discussing in detail the radiographic technique and differential diagnosis of every region of the body, but this has been admirably done in Bythell and Barclay's recent book, " X-ray Diagnosis and Treatment," which should be read by everyone interested in the subject. The book would have been of even more value to the beginner if the authors had given under each plate the details of exposure, etc., in standard units.

### General Summary.

There is no royal road to radiography. If the dissatisfied worker expects to obtain good results, simply by attending to some one hitherto neglected factor, he will be disappointed. Success can only be attained by paying minute attention to every detail, and my final advice to the beginner is: Stick to one brand of plates and one type of developer, warm the developer, work with the tube at a constant distance from the plate, use the normal current of the tube, select a tube of suitable penetration, always use a diaphragm, and, above all, *do not over-expose*.

(*To be continued.*)

* This does not apply to ordinary photography, because a negative taken through a lens is reversed with regard to both *position* and *light*; hence the positive print is normal in both respects.

## Figures and Tables

**Fig. 1. f1:**